# Arsenic in Herbal Kelp Supplements: Schenker et al. Respond

**DOI:** 10.1289/ehp.10393R

**Published:** 2007-12

**Authors:** Marc Schenker, Eric Amster, Asheesh Tiwary

**Affiliations:** UC Davis School of Medicine, University of California, Davis, California, E-mail: mbschenker@ucdavis.edu; UC Davis School of Veterinary Medicine, University of California, Davis, California

We are heartened by the feedback we have received from concerned patients, health-care professionals, herbal supplement retailers, and government health officials regarding our recent case study (Amster et al. 2007), but we certainly understand the concern from the representatives of the herbal trade industry. We would like to take this opportunity to respond to some of their comments.

In their letter, McGuffin and Dentali suggest that iodine was the cause of our patient’s symptoms, a conclusion with which we disagree. Daily intake of up to 500 μg iodine does not clinically affect the thyroid. Although it has been suggested that 1–2 mg/day is safe, there is also evidence that much higher intakes are tolerated without problems. In their comprehensive review of this subject, Backer and Hollowell (2000) concluded that “the strongest data suggest that low levels of iodine (1–5 mg/day) are safe for most people for years.” The 10th edition of the *Recommended Dietary Allowances* (National Research Council 1989) suggested a maximum allowable dietary intake of iodine of 2 mg/day for adults, and Breecher and Dworken (1986) noted that chronic toxicity develops only when intake is > 2 mg/day. Increased iodine intake (≤ 10 mg/day) may cause hypothyroidism or hyperthyroidism, but this condition is quite rare and is usually associated with underlying risk factors such as thyroiditis, subacute thyroiditis, or previously treated Graves disease. Intake of very high concentrations (18 mg to > 1 g/day) has been associated with iodine goiter (Wolff 1969).

Although we wonder how many cases of hypothyroidism are caused by irresponsible supplement use, in our case iodine toxicity is not the most likely etiology. Our patient (Amster et al. 2007) had normal thyroid function tests on two different occasions when hypothyroidism was being considered. Furthermore, she fully recovered (especially memory loss and fatigue) within 3 weeks after discontinuation of the kelp supplement. This short span of time for recovery would most likely not occur if she had iodine-induced hypothyroidism. In summary, the clinical presentation of this case was not consistent with iodine toxicity, particularly at the dose ingested. It is our clinical opinion, given the supporting clinical history and laboratory evidence, that her symptoms were more likely from the arsenic found in her kelp supplement and not from iodine. We believe this case raises legitimate concerns about arsenic toxicosis from commercially available kelp supplements and that further testing is indicated.

McGuffin and Dentali suggest that the patient is to blame for taking more than the recommended dose. We agree that the patient has ultimate responsibility to stay within the manufacturer’s guidelines, but we wonder if our patient may have been more careful in her self-prescribing if the presence of potentially toxic levels of heavy metals were included in the product labeling. This does, however, highlight a general concern of herbal medications as they are currently marketed and used: There is little oversight from prescribing health practitioners, and self-dosing commonly leads to overdosing and possible adverse herb–drug interactions (Bush et al. 2007).

McGuffin and Dentali are correct that our reporting of the dietary supplement market share was indeed misstated. We reported $178 billion from the *Nutrition Business Journal* (Anonymous 2002), but $17.8 billion is the correct figure.

Our study (Amster et al. 2007) was not intended to be a comprehensive survey of arsenic content in commercial kelp supplements, but rather to call attention to the large variability of arsenic concentrations. In his letter, Lewis suggests, without documentation, that “the arsenic most commonly found in seaweed and seafood products is relatively nontoxic,” that is, organic arsenic. In a recent study on arsenic content in ethanolic kelp and bladderwrack extracts, Krishna et al. (Krishna MVB, Brewer TM, Marcus RK, unpublished data) found that the majority (90–95%) of the arsenic present was inorganic arsenic, and only minor amounts of arsenic (5–10% of the total arsenic) were dimethyl arsenic acid. This finding suggests that the majority of arsenic in kelp supplements is the more toxic inorganic arsenic.

McGuffin and Dentali, representatives from the American Herbal Products Association, point out the difference between homeopathic and herbal therapies. We agree that there are differences, but for the purpose of our study there are obvious similarities: Both are used for medicinal purposes on a nonprescription basis and have been found to have toxic levels of heavy metals. McGuffin and Dentali are correct to point out that homeopathic medicines are regulated in a similar fashion to allopathic medicinals, as opposed to dietary supplements, which under the Dietary Supplement Health and Education Act of 1994 (DSHEA 1994) lack regulatory standards for premarket approval, good manufacturing standards, and labeling of indication (Borneman and Field 2006).

In conclusion, it was in no way our intention to attack the complementary alternative medicine community. Fortunately we all agree that the supplement industry has a responsibility to control the level of potentially harmful contaminants in their products. However, if the majority of certain herbal supplements have detectable levels of toxic metals—as is the case in our and other studies on kelp—then perhaps we should not leave the responsibility to the industry itself, but instead encourage our government to regulate these medicinals the same as all other medications, and not as dietary supplements.
